# Activation of LTRs from Different Human Endogenous Retrovirus (HERV) Families by the HTLV-1 Tax Protein and T-Cell Activators

**DOI:** 10.3390/v3112146

**Published:** 2011-11-02

**Authors:** Chirine Toufaily, Sebastien Landry, Christine Leib-Mosch, Eric Rassart, Benoit Barbeau

**Affiliations:** 1 Département des sciences biologiques, Université du Québec à Montréal, SB-R860, 2080 St-Urbain, Montréal, Québec, H2X 3X8, Canada; E-Mails: toufaily.chirine@courrier.uqam.ca (C.T.); rassart.eric@uqam.ca (E.R.); 2 Centre de recherche BioMed, Université du Québec à Montréal, SB-R860, 2080 St-Urbain, Montréal, Québec, H2X 3X8, Canada; 3 Laboratory of Genetics, The Salk Institute for Biological Studies, La Jolla, CA 92037, USA; E-Mail: slandry@salk.edu; 4 Institute of Virology, Helmholtz Zentrum München, German Research Center for Environmental Health, Institute of Virology, 85764 Neuherberg, Germany; 5 III. Medizinische Klinik, UMM-Universitätsmedizin Mannheim, University of Heidelberg, 68305 Mannheim, Germany; E-Mail: leib@helmholtz-muenchen.de

**Keywords:** HTLV-1, HERVs, Tax, CREB

## Abstract

Human endogenous retroviruses (HERVs) represent approximately 8% of our genome. HERVs influence cellular gene expression and contribute to normal physiological processes such as cellular differentiation and morphogenesis. HERVs have also been associated with certain pathological conditions, including cancer and neurodegenerative diseases. As HTLV-1 causes adult T-cell leukemia and HTLV-1-associated myelopathy/tropical spastic paraparesis (HAM/TSP) and has been shown to modulate host gene expression mainly through the expression of the powerful Tax transactivator, herein we were interested in looking at the potential modulation capacity of HTLV-1 Tax on HERV expression. In order to evaluate the promoter activity of different HERV LTRs, pHERV-LTR-luc constructs were co-transfected in Jurkat T-cells with a Tax expression vector. Tax expression potently increased the LTR activity of HERV-W8 and HERV-H (MC16). In parallel, Jurkat cells were also stimulated with different T-cell-activating agents and HERV LTRs were observed to respond to different combination of Forskolin, bpV[pic] a protein tyrosine phosphatase inhibitor, and PMA. Transfection of expression vectors for different Tax mutants in Jurkat cells showed that several transcription factors including CREB appeared to be important for HERV-W8 LTR activation. Deletion mutants were derived from the HERV-W8 LTR and the region from −137 to −123 was found to be important for LTR response following Tax expression in Jurkat cells, while a different region was shown to be required in cells treated with activators. Our results thus demonstrated that HTLV-1 Tax activates several HERV LTRs. This raises the possibility that upregulated HERV expression could be involved in diseases associated with HTLV-1 infection.

## 1. Introduction

An estimated 8% of our genome is derived from Human Endogenous Retrovirus (HERV), sequences which are resulting from integration events that took place millions of years ago. HERVs are known to have endogenized from ancestral exogenous retroviruses during primate evolution. HERVs are classified into 3 classes (class I, II, and III) based on their sequence similarities to different infectious retroviruses. Each class is divided in subgroups, based on the specificity of the tRNA primer-binding site (PBS) [1], *i.e.*, HERV-W uses the tryptophan (W) tRNA as its primer whereas HERV-K uses the lysine (K) tRNA. While most HERVs are defective and unable to produce infectious particles, some of them have retained the capacity to encode viral proteins [2]. In addition, most HERVs have lost their entire coding sequences by homologous recombination between the two LTRs, leaving solitary LTRs [3]. These LTRs remain active in their promoter and can thereby modify the expression of adjacent cellular genes.

HERV genes play an important role in many physiological events such as placental development [4,5], in which HERV-derived syncytin-1 and syncytin-2 genes seem to be the two major players by promoting the cellular fusion of trophoblasts [4,6]. On the other hand, HERVs have also been associated with several human autoimmune diseases and cancer. For instance, evidences suggest that syncytin-1 is involved in breast cancer [7,8] and multiple sclerosis [9]. In addition, a reduction in the expression of the capsid protein of HERV-W was observed in neurons and glial cells from brains of patients with schizophrenia, bipolar disorder and major depression [10], while HERV-W transcripts were more abundant in cerebrospinal fluid and plasma from patients with schizophrenia [11].

Human T-cell lymphotropic virus type-1 (HTLV-1) is the causative agents of adult T-cell leukemia (ATL) and has also been associated with a chronically progressive neuro-inflammatory disease known as HTLV-1 associated myelopathy or tropical spastic paraparesis (HAM/TSP). However, the majority of HTLV-1-infected patients remain asymptomatic throughout their lifetime. The HTLV-1 Tax protein is a powerful transactivator strongly suggested to be determinant in the development of ATL as well as HAM/TSP [12,13]. These links likely result from the capacity of this protein to activate several transcription factors such as CREB, NF-κB and SRF, which leads to upregulation or downregulation of a number of cellular genes [14–18].

Previous studies have shown that viruses such as HSV-1 and the Influenza virus could modulate HERV LTR activity [19–21]. Given that HERV overexpression has been associated with multiple sclerosis, a disease resembling HAM/TSP, we thereby tested whether the Tax protein could modulate HERV gene expression. Our results indeed confirm, that alike T cell activators, Tax significantly, yet selectively, induced LTR activity of several HERV family representatives.

## 2. Results and Discussion

### 2.1. Different HERV LTRs Are Activated upon T Cell Activation

**Figure 1.**
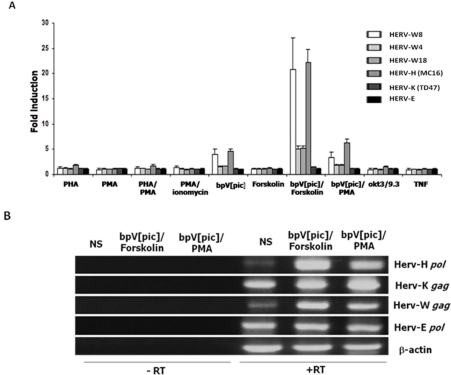
Increased expression and promoter activity of different HERVs after T-cell activation. (**A**) Reporter plasmids carrying the complete 5′LTR of different HERVs were transfected into Jurkat T-cells. At 24 h post-transfection, cells were stimulated with PHA, PMA, PHA/PMA, PMA/ionomycin, bpV[pic], Forskolin, bpV[pic]/Forskolin, bpV[pic]/PMA, OKT3(anti-CD3)/9.3 (anti-CD28) and TNFα. After stimulation (8 h), cells were lysed and measured for luciferase activity. Results are shown as fold induction relative to luciferase activity of untreated cells and are the mean of three independently treated cell samples. (**B**) Total RNA was extracted from cells treated or not with bpV[pic]/Forskolin or bpV[pic]/PMA and RT-PCR was performed for the detection of the following transcripts: the *gag* gene of HERV-K and HERV-W, the *pol* gene of HERV-H and HERV-E and β-actin. Expression levels were compared with the RNA from non-stimulated Jurkat cells (NS). Controls consisted of RT-PCR reactions conducted in the presence of RNA with no RT enzyme.

We first tested whether the activation of T lymphocytes could modulate the expression of HERVs. Different T-cell-activating agents known to activate many transcription factors in T-cells were thus first tested. Jurkat cells were transfected with luciferase reporter vectors harboring 5′LTR from different HERV families. LTRs from HERV-W4, HERV-W8, HERV W18, HERV-H (MC16), HERV-K (TD47) and HERV-E (E2) were thus tested individually for their responsiveness to T-cell activators. As shown in Figure 1A, HERV-W8 and HERV-H (MC16) LTRs were highly responsive to a combination of Forskolin and bpV[pic], a cAMP pathway activator and an inhibitor of protein tyrosine phosphatases, respectively and to the bpV[pic]/PMA combination. Both LTRs were also significantly responsive to the addition of bpV[pic] alone. While HERV-E and HERV-K representative LTRs were not activated by any tested agents, HERV-W4 and HERV-W8 presented a significant induction of LTR activity only in the presence of the Forskolin/bpV[pic] combination, although the response was more modest. To confirm these results, RNA from Jurkat cells was analyzed by RT-PCR for transcript levels of *gag* or *pol* genes from HERV-H, HERV-K, HERV-W and HERV-E families (Figure 1B). In bpV[pic]/Forskolin-stimulated cells, we confirmed that activation indeed led to an increase in HERV-W *gag* and HERV-H *pol* transcript levels when compared to untreated cells. A limited modulation of HERV-K *gag* and HERV-E *pol* expression was noted upon stimulation, again confirming the results obtained with the LTR constructs. The induction mediated by the bpV[pic]/PMA combination was also specific to HERV-H and HERV-W LTRs and was again less pronounced than the one observed with the Forskolin/bpV[pic] combination.

We and others have previously indicated that the PTP inhibitor bpV[pic] in T-cells can activate a multitude of transcription factors, such as NF-κB, NFAT, STAT, AP-1 and CREB [22–25]. Our results thereby first indicated that activation of T cells and activation of some of these transcription factors led to induction of HERV gene expression. Interestingly, we have previously demonstrated that Forskolin/bpV[pic] strongly induced the LTR of syncytin-1 (HERV-W) and syncytin-2 (HERV-FRD) genes in the choriocarcinoma BeWo cell line [4]. Other studies in T lymphocytes have indicated that HERV-H transcripts could be induced in T-cell leukemia cell lines by PHA [26] Our studies in the T-cell context are thus in line with the induction potential of HERV LTRs [27].

### 2.2. HTLV-1 Tax Activates Different HERV LTRs

**Figure 2.**
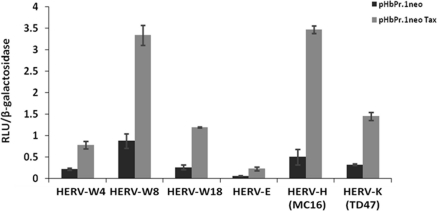
Activation of different HERV LTRs by Tax. Jurkat cells were co-transfected with a plasmid carrying the luciferase reporter gene under the control of different HERV 5′LTRs, pHβPr.1neoTax (*vs.* the empty vector pHβPr.1neo) and pRcActin-LacZ. At 24 h post-transfection, cells were harvested and assayed for luciferase activity. Results are shown as normalized luciferase values and represent the mean values of three independently transfected cell samples.

As HTLV-1 has been linked to HAM/TSP and as similar diseases are associated with elevated HERV expression, we were thus interested in looking whether the powerful Tax transactivator could positively modulate HERV expression. Since our first results indicated that specific T-cell activators could indeed upregulate HERV LTR-driven gene expression, we thereby tested whether the expression of Tax protein could also modulate HERV LTR activities. Jurkat cells were co-transfected with the different pHERV-LTR-Luc constructs along with a Tax expression vector and promoter activity was subsequently measured. As shown in Figure 2, Tax expression increased promoter activity of HERV-W (4 and 5 fold for HERV-W8 and HERV-W18 respectively) and HERV-H (MC16) (7 fold induction) but also demonstrated a significant effect on HERV-K and HERV-E LTRs.

**Figure 3.**
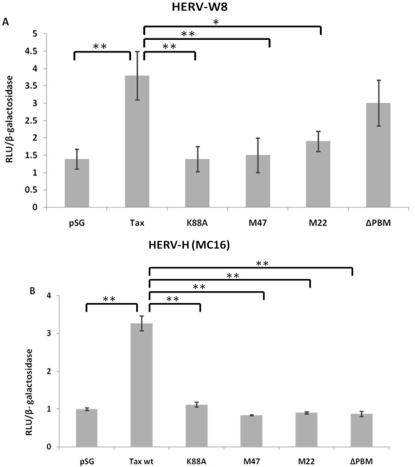

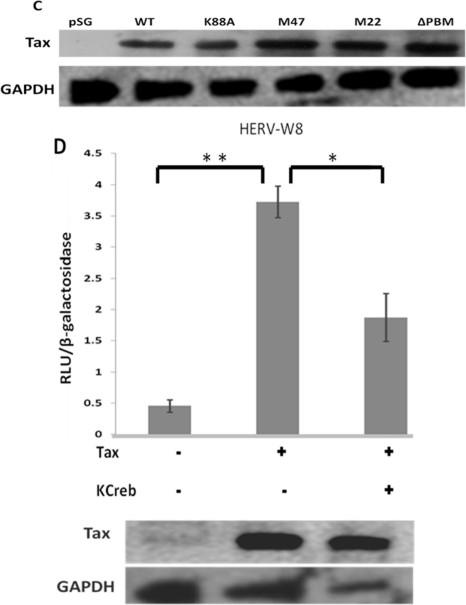
Requirement of different HTLV-1 Tax domains for HERV LTR activation. (**A**,**B**) Jurkat cells were co-transfected with a reporter plasmid carrying the HERV-W8 (**A**) or HERV-H (**B**) LTR and expression vectors for wild type or mutated version of Tax (*vs.* the empty vector). At 24 hr post-transfection, cells were harvested and measured for luciferase activity. (**C**) Using an anti-Tax antibody, Western blot analyses was carried out on extracts from Jurkat cells transfected with different Tax mutants to show equal expression of all Tax mutants and Tax WT. GAPDH is shown as a loading control (**D**) Jurkat cells were co-transfected with the luciferase reporter construct under the control of the HERV-W8 LTR in the presence or absence of a Tax expression vector (*vs.* the empty vector) and the expression vector for KCREB (*vs.* empty vector). All transfections were conducted in the presence of pRcActin-LacZ for normalization. Results are shown as normalized luciferase values and represent the mean values of three independently transfected cell samples. A western blot analysis using an anti-Tax antibody was carried out to confirm equal expression of Tax between samples. GAPDH is shown as a loading control.*, P < 0.05;**, P < 0.01.

In order to better understand how Tax could activate transcription of HERV LTRs, the pHERV-W8 LTR-Luc construct was co-transfected with expression vectors for different Tax mutants. Both Tax K88A (Tax deficient for CBP/p300 recruitment) and Tax M47 (deficient for CREB activation) showed a limited induction of luciferase activity in transfected Jurkat cells when compared to wild-type Tax (Figure 3A). TaxM22, a NF-κB activation-defective Tax also was not able to transactivate the LTR of HERV-W8 whereas inactivation of the PDZ binding motif had a more limited effect on the Tax activation potential, which was not statistically significant. To further confirm the impact of the various Tax mutants on HERV LTR activation, similar transfections were repeated in Jurkat cells with the HERV-H (MC16) LTR-driven luciferase gene. As depicted in Figure 3B, all Tax mutants (including the ΔPBM mutant) were significantly less efficient in activating the HERV-H LTR than was wild-type Tax. Since two Tax mutants are deficient for CREB-specific activation, we next tested whether CREB is involved in the activation of HERV-W using a CREB-specific dominant negative mutant termed Killer CREB (KCREB). Jurkat cells were thus co-transfected with wild-type Tax and KCREB expression vectors along with the HERV-W8 LTR-driven luciferase reporter vector. When KCREB was expressed, Tax-induced HERV LTR activity was significantly reduced (Figure 3C). In the above experiments, levels of Tax expression were controlled for each transfection condition and demonstrated comparable levels as determined by Western blot analyses (Figures 3C,D).

A limited number of studies have looked at possible association between viruses and HERV expression. HSV-1 and the influenza virus have been shown to modulate LTR activity of HERV-W while HERV-K LTR activity was positively modulated by the influenza A/WSN/44 strain [19–21]. A number of studies have also highlighted an increase in the expression of mostly HERV-K family members in HIV-1-infected patients, thereby leading to a CTL response toward HERV proteins [28–30]. An *in vitro* study has further shown that HIV infection leads to increase in HERV-K expression [31]. We therefore present for the first time data indicating that HTLV-1 through Tax also mediates the activation of HERV LTRs. Interestingly, based on the data with the Tax mutants, multiple transcription factors seem to mediate the activation of HERV LTRs. Although there are similarities between the response of the different HERV LTRs to Tax expression, a difference in the response of HERV-W and HERV-H LTRs to the Tax ΔPBM mutant was observed. This likely reflects differences in the composition of responsive elements in their promoter region.

### 2.3. HERV-W8 LTR-Responsive Regions to Tax and T-Cell Activators Are Different

**Figure 4.**
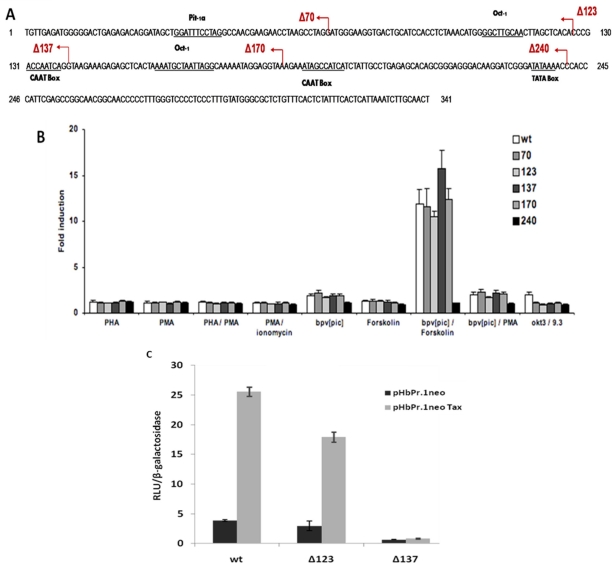
Identification of HERV-W8 LTR regions required for activation by T cell activators and by Tax. (**A**) Tested 5′ deletion mutants of the HERV-W8 LTR. (**B**) Reporter plasmids carrying the complete LTR and 5′ deletion mutants were transfected into Jurkat cells along with pRcActin-LacZ. At 24 h post-transfection, cells were stimulated with PHA, PMA, PHA/PMA, PMA/Ionomycin, bpV[pic], Forskolin, bpV[pic] /Forskolin, bpV[pic] /PMA, OKT3/9.3 and TNFα. After 8 h of stimulation, cells were harvested and assayed for luciferase activity, which was normalized against β-galactosidase activity. Results are shown as fold stimulation relative to luciferase activity of untreated cells. (**C**) Jurkat cells were co-transfected with a plasmid carrying the luciferase reporter gene under the control of different HERV 5′ LTRs, pHβPr.1neoTax (*vs.* the empty vector pHβPr.1neo) and pRcActin-LacZ. At 24 h post-transfection, cells were harvested and assayed for luciferase activity. Results are shown as normalized luciferase values and represent the mean values of three independently transfected cell samples.

To identify the region responsive to both T-cell activators and Tax, 5′ deletion mutants were generated from the HERV-W8 LTR construct (Figure 4A). Jurkat cells were first transfected with these deletions mutants and the full-length LTR construct and stimulated with the different T-cell activators. As indicated above, the HERV-W8 LTR was mostly induced in Jurkat cells stimulated with a combination of Forskolin and bpV[pic]. A dramatic decrease in the induction was noted exclusively with the Δ240 mutant (Figure 4B). Deletion mutants were subsequently co-transfected with the wild-type Tax expression vector in Jurkat cells and compared to cells transfected with the empty vector (Figure 4C). Interestingly, a significant reduction in basal and Tax-mediated LTR activation was observed with the 5′ deletion mutant Δ137. Although basal activity was affected, fold induction was nonetheless greatly decreased, thereby showing a distinct region of responsiveness between Tax and our tested T-cell activators.

Previous studies have identified several elements acting on basal and induced HERV LTR-driven gene expression, which can bind specific transcription factors. For HERV-W and HERV-H, previous reports had indeed indicated that HSV infection mediates LTR activation through Oct-1- and AP-1-binding sites, respectively [19,20]. More specific studies focused on a HERV-W representative encoding for syncytin-1 have revealed that several transcription factors were acting on basal and cAMP-mediated LTR activation in trophoblast cells, such as GCMa, Sp1, GATA transcription factors and other potential transcription factors [5,32–34]. A potential NF-κB-binding site has also been identified for its importance in TNF-α-mediated activation of syncytin-1 expression in astrocytes [35]. Our results suggest that different transcription factors act upon the induction of HERV-W8 LTR by T-cell activators and Tax. We are currently conducting experiments to more precisely identify these LTR regions.

## 3. Experimental Section

### 3.1. Plasmids

pBL-based constructs containing different HERV LTRs (HERV-W4, HERV-W8, HERV-W18, HERV-E2, HERV-H (MC16), and HERV-K (TD47) inserted upstream of the firefly luciferase reporter have been previously described [36]. The Tax expression vector pHβPr.1neoTax and the empty vector pHβPr.1neo were generously provided by Dr. M. Nakamura (Tokyo Medical and Dental University, Tokyo, Japan) [37]. Vectors expressing wild-type and mutated Tax [38] were kindly provided by Dr. J.M. Mesnard (Université Montpellier 1, Montpellier, France). The CREB dominant negative mutant KCREB and the control empty vector [39] were provided by Dr. R.H. Goodman (Vollum Institute for Advanced Biochemical, Research, Portland, OR, USA). The pRcActin-LacZ vector contains the β-galactosidase gene under the control of the β-actin.

### 3.2. Generation of Deletions Mutants by Exonuclease III

The pHERV-W8 LTR-Luc construct was first digested with BstX1 and BamHI and subsequently incubated in the presence of exonuclease III at 37 °C. At 15 s intervals, an aliquot was taken and added to a tube containing S1 nuclease. At the end of the time course, the S1 nuclease reaction was completed at 30 °C. Each samples were heat inactivated and after religation, transformed in DH5α. Sequencing of plasmid DNA from resulting colonies was conducted for positioning the resulting 5′ end of the LTR. Five deletion mutants were chosen for subsequent experiments.

### 3.3. Transfection and Assay for Luciferase Activity

Transfection of Jurkat cells was carried out by electroporation for 24 hours with a total of 15 μg of DNA (250 V and 950 μF) according to the Hughes and Pober’s protocol (Hughes and Pober, 1996). Transiently transfected cells were seeded at a density of 10^6^ cells/well in 6-well plates and left unstimulated or treated for 8 h with PHA (3 μg/mL), PMA (20 ng/mL) (Sigma), ionomycin (1 μM) (Calbiochem), anti-CD3 antibody (clone OKT3) (3 μg/mL), anti-CD28 antibody (clone 9.3) (1 μg/mL), Forskolin (100 μM) (BioMol, Plymouth Meeting, PA) and bpVpic (10 μM) in a final volume of 3 mL. Cells were then lysed in a 1× lysis buffer (25 mMTris phosphate, pH 7.8, 2 mM DTT, 1% Triton X-100, 10% glycerol). Luciferase activity was determined as follows. After a freeze/thaw cycle, 25 μL of cellular extract was transferred to a 96-well luminometer plate and luciferase activity was quantified on a Dynex MLX microplate luminometer (MLX; Dynex Technologies, Chantilly, VA, USA) following a single injection of a luciferase buffer [137 mM NaCl, 20 mM tricine, 1.07 mM(MgCO_3_)_4_·Mg(OH)_2_·5H_2_O, 2.67 mM MgSO_4_, 0.1 mM EDTA (ethylenediaminetetraacetic acid), 220 μM coenzyme A, 4.7 μM D-luciferin potassium salt, 530 μM ATP, 33.3 mM DTT]. β-galactosidase activity was measured using the Galacto-Light™ kit (Applied Biosystems, Bedford, MA, USA) according to manufacturer’s instructions. Luciferase activity was calculated in terms of relative light units (RLU) and represents the mean ±SD of three transfected samples normalized for β-galactosidase activity. Fold inductions were calculated by dividing the values of activated samples by values of non-stimulated samples.

### 3.4. Total RNA Extraction and Semi-Quantitative RT-PCR

Total RNA was isolated from Jurkat cells using the Trizol reagent (Invitrogen Canada Inc). Prior to RT, total RNA was treated with TurboRNAse-Free DNAse (Ambion, Austin, TX, USA) for 5 min at 70 °C. RNA (1 μg) was then incubated in the presence of oligo(dT) (25 ng/μL), 10 mM DTT, 100 mM dNTP (deoxynucleotide triphosphate), SuperScript reverse transcriptase (10 U) (Invitrogen Canada Inc.), and SUPERase-In (20 U) at 42 °C for 50 min. Aliquots from the RT reactions were then PCR-amplified in the presence of 1U Taq DNA polymerase (New England Biolabs, Pickering, Canada), 1× ThermoPol buffer, 100 μM dNTP, and 15 μM of each primer. Primers used for HERV-H *pol* cDNA amplification were 5′-CCTTTATTACCCAATCTGCTCCCGA(CT)AT-3′ (forward) and 5′-TTTAGTGGTGGACAGTCTCTTTTCCA(AG)TG-3′ (reverse).

For HERV-K *gag* cDNA amplification, the primers were 5′-TCCCCTTGGAATACTCCTGTTTT(CT)GT-3′ (forward) and 5′-CATTCCTTGTGGTAAAACTTTCCA(CT)TG-3′ (reverse).

For HERV-W *gag* cDNA, the primers were 5′-GGCCAGGCATCAGCCCAAGACTTG-3′ (forward) and 5′-CTTTAGGGCCTGGAAAGCCACT-3′ (reverse), as for HERV-E *pol* cDNA amplification, the primers were 5′-CATCAACCTACTTGGGATTGTCA(AG)CA-3′ (forward) and 5′-CAATGACCTTTTTCTTTACAGTAGGC(AG)CA-3′ (reverse).

For RT-PCR analyses of β-actin mRNA, the primers 5′-CGTGACATTAAGGAGAAGCTG-3′ (forward) and 5′-CTCAGGAGGAGCAATGATCTT-3′ (reverse) were used. PCR conditions were as follows: a first step of denaturation at 95 °C for 3 min. followed by 35 cycles of denaturation (94 °C for 30 s), annealing (60 °C for 15 s) and elongation (72 °C for 12 s).

### 3.5. Western Blot Analyses

Western blot analyses from total protein isolated from transfected Jurkat cells were performed. 24 h post-transfection, cells were washed with PBS 1× and lysed with lysis buffer (50 mM Tris-HCl, pH 7.4, 120 mM NaCl, 5 mM EDTA, 0.5% Nonidet P-40, 0.2 mM Na_3_VO_4_, 1 mM dithiothreitol, 1 mM phenylmethylsulfonyl fluoride) in the presence of protease inhibitors (Complete, Roche Applied Science), and incubated on ice for 30 minutes. Cell debris were pelleted by centrifugation for 10 min at high speed. Protein concentrations were quantified with the bicinchoninic acid (BCA) protein assay (Thermo Fisher Scientific Inc., Rochester, NY, USA). Extracts were migrated on a 12% sodium dodecyl sulfate polyacrylamide gel electrophoresis (SDS-PAGE) and transferred on a polyvinylidene fluoride (PVDF) membrane (Millipore). Membrane was blocked with 5% Bovine Serum Albumin (BSA) and incubated with anti-Tax antibody (dilution, 1/100) (or a polyclonal anti-glyceraldehyde-3-phosphate dehydrogenase (GAPDH) antibody (1/5000; Santa Cruz, CA, USA). Membranes were further incubated with a horseradish peroxidase-coupled anti-mouse antibody (1/10,000) (Amersham Biosciences, Buckinghamshire, UK), and signals were detected using the BM chemiluminescence blotting substrate (Roche Diagnostics). Membranes were subsequently exposed on an ECL high performance chemiluminescence film (Amersham Biosciences). Antibodies from the HTLV-I Tax hybridoma 168A51-42 (Tab176) was obtained from Dr. J.M. Mesnard [40].

### 3.6. Statistical Analyses

All experiments were performed in triplicates. Results are expressed as the mean+SEM and statistically analyzed using a 2-tailed Student t test for 2-group comparisons.

## 4. Conclusions

We herein have demonstrated that LTRs from different HERV families can both be activated by the combination of T-cell activation agent bpV[pic]/Forskolin and bpV[pic]/PMA and by the Tax transactivator. Furthermore, we identified a Tax-responsive region different from the region responsive to T-cell activators suggesting the implication of different transcription factors. Indeed the use of the various Tax mutants reveals that each of them affects this LTR activation at different degrees. Through the CREB dominant negative mutant, our results argue that members of the CREB/ATF family are playing a role in the upregulation of the LTR activity. The HTLV-1 Tax protein is a powerful transactivator, capable of inducing many cellular genes with its activation domains. It is thereby not surprising that it also acts on the HERV-W LTRs, which are known to be upregulated by inducing agents such as Forskolin and bpV[pic] in human trophoblasts and by HSV-1 and Influenza virus infection [4,20,21]. We conclude that Tax can modulate these LTRs in Jurkat cells by activating CREB and possibly NF-κB, which can positively regulate the transcription of the HERVs genes or other cellular genes in proximity. Transcription factors mediating the upregulation of HERV LTRs by both Tax and T-cell activators are likely different and we are currently working on their identification.

Since our results indicate that Tax can modulate the expression of HERVs, a link between HTLV-1-associated diseases and HERV dysregulation is an interesting speculation. Indeed, upregulation of HERV gene expression has been associated with various inflammatory and autoimmune diseases such as multiple sclerosis and arthritis [9,41]. Interestingly, HTLV-1-associated diseases HAM/TSP and HTLV-1-associated arthropathy have been shown to be very similar to these latter diseases. In addition, activation of HERV LTR nearby proto-oncogenes may constitute a mechanism by which Tax could promote cell transformation via HERV sequences. Alternatively, induced expression of near full-length HERV proviral DNA could generate potential substrates for reverse transcriptase activity. Newly synthesized proviruses could then reintegrate the host genome in infected cells and contribute to genomic instability.

More advanced studies are needed to clearly determine if HERV expression is increased in HTLV-1-infected patients and modulated during the course of HTLV-1-induced pathologies. Furthermore, in this study, we have focused on the modulation of HERV-LTR by the viral protein Tax of HTLV-1. Clearly, other viral proteins such as HBZ could impact on the extent of Tax-mediated HERV LTR activation or could affect HERV LTR activation. These experiments, as well as the analysis of HERV expression in HTLV-I-infected cells are currently ongoing. These studies will indicate whether HERVs could become possible new disease markers in HTLV-1-infected patients.
